# Halogen-Doped Carbon Dots: Synthesis, Application, and Prospects

**DOI:** 10.3390/molecules27144620

**Published:** 2022-07-20

**Authors:** Kun Luo, Yanmei Wen, Xinhuang Kang

**Affiliations:** Faculty of Chemistry and Environment Science, Guangdong Ocean University, Zhanjiang 524088, China; lk13272906984@163.com

**Keywords:** carbon dots, halogen doping, fluorescence, applications, sensors, biomedicine

## Abstract

Carbon dots (CDs) have many advantages, such as tunable photoluminescence, large two-photon absorption cross-sections, easy functionalization, low toxicity, chemical inertness, good dispersion, and biocompatibility. Halogen doping further improves the optical and physicochemical properties of CDs, extending their applications in fluorescence sensors, biomedicine, photocatalysis, anti-counterfeiting encryption, and light-emitting diodes. This review briefly describes the preparation of CDs via the “top-down” and “bottom-up” approaches and discusses the preparation methods and applications of halogen (fluorine, chlorine, bromine, and iodine)-doped CDs. The main challenges of CDs in the future are the elucidation of the luminescence mechanism, fine doping with elements (proportion, position, etc.), and their incorporation in practical devices.

## 1. Introduction

The term “carbon dots” (CDs) usually refers to carbon or graphene quantum dots (CQDs or GQDs, respectively), carbon nanodots, or polymer dots [1]. As a new type of carbon nanomaterial, CDs are defined as dispersed spheroidal carbon particles less than 10 nm in diameter with fluorescence properties [2]. In 2004, fluorescent carbon was first isolated by the Scrivens group during the purification of single-walled carbon nanotubes [3]. In 2006, these fluorescent carbon nanoparticles were first referred to as CDs [4].

**Figure 1:**
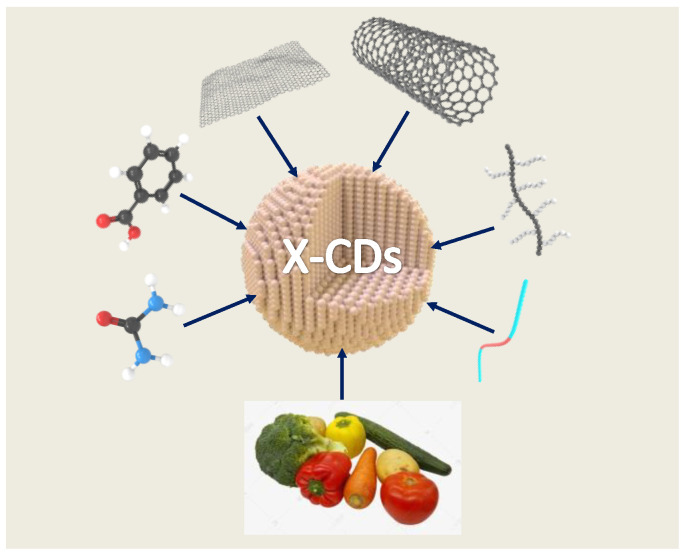
Various raw material sources used in the synthesis of CDs including small molecule compounds, polymers, carbon materials, fruits, and vegetables.

The CDs core is dominated by sp^2^-hybridized carbon atoms, and there are abundant surface functional groups [5,6]. The internal electronic structures include σ → σ*, σ → π*, π → π*, n → π*, and n → σ* electron transitions, which affect the physicochemical properties of CDs, especially their optical properties [7,8]. The design and synthesis of CDs have been extensively studied owing to the numerous advantageous characteristics of CDs, such as high luminous efficiency, stability, and much greater optical stability than organic dyes and semiconductor quantum dots [9,10,11]. Moreover, they have low toxicity, good biocompatibility, excellent photostability, and tunable fluorescence [12,13,14,15]. Under different excitations, CDs possessing different particle sizes, elemental doping contents, and surface functional groups can emit different types of fluorescence [16,17]. The preparation methods of CDs are diverse, and they have been prepared using graphene, carbon nanotubes, organic small molecules, oligomers, fruits, and vegetables as raw materials (Figure 1).

Their highly advantageous optical properties endow CDs with considerable research value [18,19]. However, traditional preparation methods yield CDs with several drawbacks, such as low fluorescence quantum yields (QY), low visible-light utilization, and short emission wavelengths, significantly limiting their applications. To overcome these shortcomings, surface passivation and heteroatomic doping have been performed on CDs. However, surface passivation often involves tedious steps and toxic chemicals and changes the location and number of the original functional groups used for sensing and analysis [20,21]. Heteroatomic doping involves introducing non-metallic atoms or metal ions to CDs to change their energy-gap structure and generate different intrinsic properties [22]. The latter is one of the most effective, extensive, and sensitive methods for controlling the optical and electronic transport properties of CDs [23,24]. Recently, non-metallic atom doping of CDs has become common, including nitrogen [25,26], phosphorous [27,28], sulfur [29,30], and silicon [31,32]. Doping changes the distributions and surface chemical structures of the different carbon hybrids, improving the optical properties of CDs and facilitating new applications.

This review primarily discusses halogen-doped CDs preparation methods and performance. Specific applications in anti-counterfeiting encryption, environmental monitoring, biosensing, gene detection, drug delivery, and biological imaging are also summarized. Hence, a theoretical basis for future CDs research and development is established.

## 2. CDs Preparation Methods

**Figure 2:**
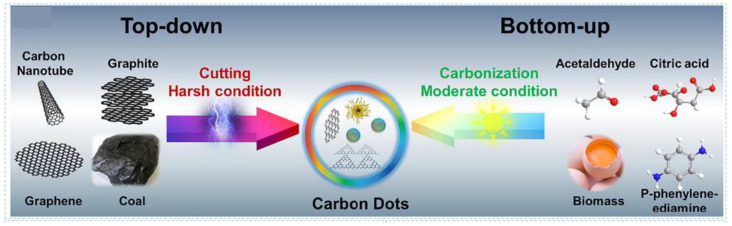
Top-down and bottom-up method approaches to the synthesis of CDs based on their raw materials. Reprinted/adapted with permission from Ref. [54]. Copyright 2021, copyright Elsevier.

Since their discovery, the efficient preparation of CDs and improving their QY has remained topical. Current CDs synthesis methods can be divided into top-down [3,25,33,34,35,36,37] and bottom-up [38,39,40,41,42,43,44,45,46,47,48,49,50,51,52,53], as summarized in Figure 2.

### 2.1. Top-Down Approach

**Figure 3:**
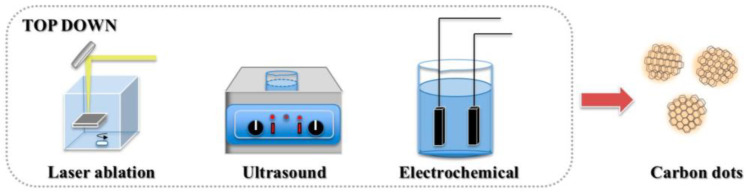
Top-down methods for the synthesis of CDs [55].

In the top-down method, CDs are formed via physical or chemical means through the disassembly of large amounts of carbon-rich materials (e.g., graphene, carbon nanotubes, candle ash, and carbon black). Laser ablation [35], arc discharge [3], electrochemical synthesis [25], and chemical oxidation reactions [36] are usually employed (Figure 3). However, owing to broken-site randomness, strict control of the size and structure of CDs is difficult. The top-down method generally involves complex processes and expensive equipment and has a low yield; thus, it is unsuitable for large-scale production and practical applications.

#### 2.1.1. Laser Ablation

In 2006, Sun et al. [4] prepared CDs by laser ablation for the first time. The product obtained from the laser ablation of carbon raw material was refluxed in dilute nitric acid for 12 h, and then passivated with PEG1500N (amine-sealed polyethylene glycol) and poly (propyl ethylene imide—polyethylene imide). Finally, the CDs was treated with acid to produce a bright fluorescence. CDs obtained via laser ablation is not uniform in particle size and requires relatively specialized equipment, making it not suitable for industrial production.

#### 2.1.2. Arc Discharge

The first CDs discovered were synthesized using the arc discharge method. Xu et al. [3] oxidized arc-treated soot with 3.3 N HNO_3_ and extracted the resulting black suspension with a NaOH solution to yield CDs with increased hydrophilicity. A fast-moving strip of highly fluorescent material with carboxylated carbonaceous spheres was separated from the black suspension by gel electrophoresis [3]. The CDs obtained by this method exhibited good fluorescence, but their particle size was non-uniform and the yield was very low, which is not suitable for mass production.

#### 2.1.3. Electrochemical Synthesis

CDs can be prepared via the electrochemical method in higher yield and more uniform particle size under mild reaction conditions and with less damage to the environment. However, specialized equipment is required and fewer raw materials are suitable for this method. Yang et al. [25] coated nitrogen-doped graphene on the surface of a carbon electrode and directly synthesized nitrogen-doped graphene quantum dots (N-GQDs) in a yield of 25%. The N-GQDs were rich in hydroxyl, carboxyl, and nitro groups, and exhibited a QY of 10%.

### 2.2. Bottom-Up Approach

**Figure 4:**
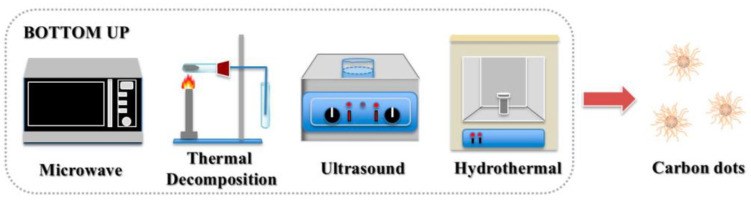
Bottom-up synthesis of CDs [55].

In bottom-up methods, organic small molecules or oligomers (e.g., citric acid, glucose, polyethylene glycol, urea, and ionic liquid) are used as CDs synthesis precursors. Hydrothermal reactions [45], solvothermal reactions [47], microwave pyrolysis [48], ultrasonic treatment [50], templates [56], and chemical vapor deposition [51] have been employed (Figure 4). These methods are simple and cheap, and doping is straightforward. Moreover, because obtained CDs are comparable to traditional semiconductor quantum dots, these synthetic routes are attracting increasing amounts of attention.

#### 2.2.1. Hydrothermal or Solvothermal Synthesis

Hydro/solvo-thermal synthesis is a very common method, and is simple, safe, and efficient, and more environmentally friendly. He et al. [45] produced CQDs via hydrothermal synthesis using lemon juice as a carbon source that exhibits bright blue-green light emission under ultraviolet or blue light irradiation. Zhang et al. [47] synthesized differently colored CQDs by solvent-thermal synthesis using *o*-phenylenediamine as raw material and trichloromethane as solvent.

#### 2.2.2. Microwave Pyrolysis

Microwave reactions offer an efficient method to synthesize CDs, with greatly shortened reaction times. The general microwave process includes the pyrolysis and surface functionalization of a carbon source. For example, Mahmoud et al. [48] synthesized CQDs with excellent fluorescence characteristics by microwave pyrolysis of an aqueous starch solution for 10 min.

## 3. Halogen-Doped CDs

In 2013, Zhou et al. [57] developed a simple and effective synthesis method for halogen (chlorine (Cl), bromine (Br), iodine (I))-doped CQDs with prominent fluorescence under UV-lamp irradiation. The synthesized Cl-CQDs were used as reaction intermediates and were selectively quenched by ferric iron (Fe^3+^), thus introducing concepts for future CQDs halogen functionalization.

Because of the large electronegativity difference between carbon and halogens (X = F, Cl, Br, or I), halogen doping impacts the photoluminescence (PL) and pH stability of CDs. Moreover, it inhibits the reactivity of irradiation-generated singlet oxygen. Therefore, halogen doping of CDs improve their practical applicability. In addition, X-CDs can also be used to modify amorphous cobalt phosphate compounds [58], that owing to the different properties of various X-CDs, enables the fine-tuning amorphous cobalt phosphating compounds for targeted applications.

### 3.1. Fluorine Doping

#### 3.1.1. Single-Fluorine Doping

**Figure 5:**
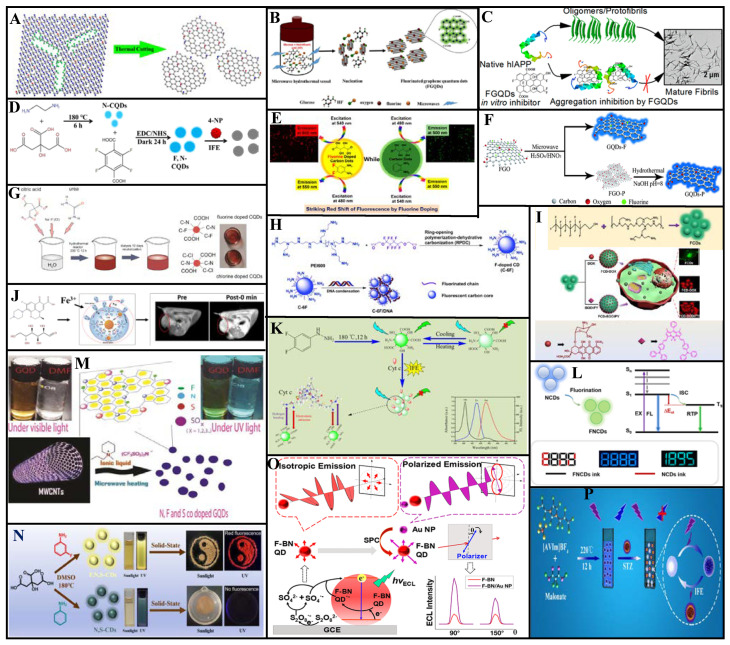
Synthesis and applications of F-doped CDs [59,61]. (**A**) F-GQDs were prepared using pyrolytic fluorinated graphite at 810 K. (**B**) HF was used as the dopant, and F-GQDs were synthesized by the microwave assisted hydrothermal method and (**C**) used to inhibit hIAPP polymerization. (**D**) Schematic diagram of FN-CQDs synthesis and used for 4-NP detection. (**E**) Prominent red shift in the fluorescence by F-doping used for cell imaging. (**F**) FGO synthesizes F-GQDs as the precursor. (**G**) Synesthetic route of the F-CQDs. (**H**) F-CDs were prepared using the RPDC method and polymerized with a DNA molecular chain. (**I**) Schematic of F-CDs, F-CDs-DOX, and F-CDs-BODIPY fabrication and the cellular uptake of F-CDs-DOX. (**J**) FN-CQDs were synthesized with levofloxacin and glucose by microwave assisted synthesis, and combined with Fe^3+^ as the contrast agent. (**K**) Illustration of F, N-CDs synthesis, and the detection principle for Cyt c and temperature. (**L**) Formation of RTP is related to the cross-stable triple excited states in the system, and FN-CDs are used as the anti-counterfeiting fluorescent ink. (**M**) High QY (70%) NFS co-doped GQDs were synthesized using the microwave method with a customized ionic liquid base. (**N**) NFS co-doped CDs were synthesized by the solvothermal reaction of citric acid and m-fluoroaniline in the presence of DMSO. (**O**) Polarized SPC-ECL mechanism of the F-BN QDs. (**P**) N, B, and F co-doped CDs were prepared by 1-allyl-3-vinyl imidazolium tetrafluoroborate, and sulfathiazole was detected based on internal filtration effect. Reprinted/adapted with permission from Ref. [63]. Copyright 2015, copyright John Wiley. Reprinted/adapted with permission from Refs. [65,70]. Copyright 2018 and 2015, copyright RSC. Reprinted/adapted with permission from Refs. [65,68,69,71,73]. Copyright 2020 and 2021, copyright Elsevier. Reprinted/adapted with permission from [60,62,64,66,67,72]. Copyright 2017, 2019, 2020 American Chemical Society.

Fluoride doping improves the optical properties of CDs and confer unique magnetic properties. Feng et al. [59] reported the top-down synthesis of uniform fluorinated GQDs (F-GQDs; average particle size: 5.11 nm, QY: 6.3%, F/C atomic ratio: ca. 0.03) via pyrolysis through thermal cutting of highly fluorinated graphene with a large number of structural defects at 810 K (Figure 5A). These F-GQDs exhibit excitation wavelength-dependent properties with multicolor PL from blue to green. In addition, the many sp^3^-type defects and magnetic zigzag edges produced by thermal cutting provide the F-GQDs with strong spin-half paramagnetism (*M_s_* = 0.680 emu/g), with a localized spin number of ~7.33 × 10^19^ g^−1^. These F-GQDs have multimodal capability, multicolor luminescence, and strong paramagnetism considerable applicability in materials science.

Yousaf et al. [60] synthesized F-GQDs (average particle diameter: 2.38 nm) via microwave-assisted hydrothermal synthesis using glucose as a precursor and hydrofluoric acid as a dopant (Figure 5B). These F-GQDs exhibit good water dispersion and excellent fluorescence and were employed as a therapeutic agent against human islet amyloid polypeptide (hIAPP) aggregation. They also inhibited conformational transition of the peptides from the natural structure to a *β*-sheet (Figure 5C). With increasing F-GQDs concentration, the hIAPP-induced INS-1 cytotoxicity was inhibited. Thus, these F-GQDs are biomedically significant for amyloidosis prevention and treatment.

#### 3.1.2. Fluorine and Nitrogen Co-Doping

Nitrogen doping significantly improves the QY of CDs and optimizes their structures and luminescence. As shown in Figure 5D, Feng et al. [61] produced N-CDs via hydrothermal treatment of citric acid and ethylenediamine. They then added tetrafluoroterephthalic acid to the N-CDs solution. After 24 h dark treatment with N-ethyl-N-(3-(dimethyl amino propyl) carbodiimide) and N-hydroxysuccinimide, they obtained FN-CDs with QY up to 58.9%. Fluorescence quenching was caused by interactions between the FN-CDs functional groups and 4-nitrophenol (4-NP) and the inner filter effect. When applied with liquid–liquid microextraction, these FN-CDs could detect 4-nitrophenol with a 15 nM limit of detection (LOD).

Zuo et al. [62] prepared FN-CDs possessing dual-wavelength fluorescence emission via the solvothermal method using 4,5-difluorobenzene-1,2-diamine and tartaric acid as precursors. Efficient cell imaging was achieved because these FN-CDs emit strong red fluorescence under 540–580 nm excitation. The QY of FN-CDs at 550 and 600 nm emission wavelengths were 31% and 14%, respectively, exceeding those of N-CDs. Moreover, the FN-CDs were used as fluorescent probes for Ag^+^ detection (Figure 5E).

Sun et al. [63] developed a top-down synthesis strategy for FN-GQDs (QY: 7.5%) by subjecting fluorinated graphene oxide nanosheets to microwave-assisted hydrothermal treatment under acidic conditions (Figure 5F). Fluorine altered the energy gaps between the highest occupied and lowest unoccupied molecular orbitals of the graphene, reduced the π-electron density, and formed a protective GQDs surface shell. Thus, F-doping improved the photo and pH stability GQDs. Moreover, FN-GQDs are suitable for long-term cellular imaging and biosensing owing to their good biocompatibility. The authors enhanced fluorescence around the nuclei of cervical carcinoma HeLa cells by treating them with FN-GQDs over a 4 h incubation period; the sufficient fluorescence stability across 60 min facilitated long-term biological imaging. Thus, FN-GQDs can label cell membranes and cytoplasm.

Using sodium fluoride, urea, and citric acid as precursors in a one-step hydrothermal synthesis, Marković et al. [64] prepared FN-CQDs (average particle size: 5.2 nm, QY: 15.1%) with good antioxidant activity and higher-efficiency energy transfer than CQDs. These FN-CQDs are suitable as fluorescent probes for cell imaging (Figure 5G). Notably, the charge transfer resistance and transport bandgap of the doped CQDs film are two orders of magnitude higher and 2 eV larger than those of un-doped CQDs film, respectively.

Luo et al. [65] prepared FN-CDs (particle size distribution: 1.5–3.5 nm) using a ring-opening polymerization and dehydration carbonization method with polyethyleneimine and fluorinated diglycidyl ether as precursors (Figure 5H). The prepared FN-CDs emit blue fluorescence under UV-light irradiation. Notably, these FN-CDs have high surface positive charge density and effectively condense charged DNA into appropriately sized nanoparticles through electrostatic interaction, thereby promoting gene transfection efficiency.

Gao et al. [66] prepared nucleus-targeting FN-CDs that are promising for drug and dye delivery applications. For example, through the assembly of FN-CDs and doxorubicin or boron dipyrromethene, nanocomposites facilitating cellular uptake and delivery can be constructed. FN-CDs-based nanomaterials have considerable potential for nucleus-targeted bioimaging and cancer therapy (Figure 5I).

Wang et al. [67] obtained highly hydrophilic FN-CDs from glucose and levofloxacin through microwave-assisted thermal decomposition and formed a complex using the strong affinity between their surface groups and Fe ions. These complexes constitute novel, safe, efficient, and promising contrast agents for *T*1-weighted magnetic resonance imaging because of their extremely low toxicity, high *r*1 relaxation, strong light luminescence, and low synthetic cost (Figure 5J).

Zhu et al. [68] prepared FN-CDs (QY: 16.9%) via solvent heat treatment of 3, 4-difluorophenylhydrazine. Their optimal emission wavelength is 530 nm with yellow-green fluorescence, and these FN-CDs can be used as bio and temperature sensors. Linear and reversible fluorescence intensity variations occur in the range 25–60 °C. The fluorescence is quenched by cytochrome C based on the inner filter effect. Good linear correlation between the fluorescence quenching efficiency and cytochrome C concentration occurs in the 0.5–25 µM range, with a 0.25 µM LOD, 93.14–110.40% recovery, and high sensitivity (Figure 5K).

As shown in Figure 5L, Liu et al. [69] prepared FN-CDs with room-temperature phosphorescence (RTP) characteristics. Aqueous FN- and N-CDs solutions were used as ink and interference ink, respectively, for encoding/reading experiments, and their patterns yielded the same blue fluorescence under 365 nm UV light irradiation. When the UV lamp was deactivated, the N-CDs ink interference image extinguished, and the FN-CDs ink phosphorescence image gradually appeared because of the inherent emission difference between fluorescence and RTP. These FN-CDs can be used for high-resolution information storage and anti-counterfeiting.

#### 3.1.3. Nitrogen, Fluorine, and Sulfur Co-Doping

Kundu et al. [70] synthesized N, F, and S co-doped GQDs (NFS-GQDs) using multiwalled carbon nanotubes in a customized ionic liquid via a simple one-step microwave method (Figure 5M). These NFS-GQDs have excitation-independent optical properties. When irradiated with 375 nm light, their maximum emission wavelengths are 409 and 435 nm, which can be attributed to the small energy gap between their singlet and triplet states. Thus, the combined use of ionic liquid and microwave assistance facilitates rapid synthesis and improves the QY (70%) of NFS-GQDs through the synergistic effects of defect clusters and dopants.

Ding et al. [71] synthesized NFS-CDs with red-emissive solid-state fluorescence through a facile, one-pot solvothermal approach (Figure 5N). Because F-induced defect traps exist on the surfaces/edges, these NFS-CDs have higher QY (3.17%) than NS-CDs and greater emission wavelengths. Interestingly, solid-state NFS-CDs exhibit temperature-sensitive behavior at 80–420 K, with maximum fluorescence intensity at 120 K. Thus, these NFS-CDs have potential as temperature fluorescence sensors and solid light-emitting devices with broad temperature adaptability.

#### 3.1.4. Fluorine, Boron, and Nitrogen Co-Doping

Liang et al. [72] synthesized F-doped boron nitride quantum dots (F-BN QDs) using H_3_BO_3_, C_3_H_6_N_6_, and NH_4_F as precursors through a microwave-assisted hydrothermal synthesis pathway. These F-BN QDs have unique characteristics, e.g., photoluminescent blue light and electroluminescent yellow light. A polarized electrochemiluminescence (ECL) biosensor was established using the F-BN QDs and gold nanoparticles to detect the K-ras gene in a 0.1 fM–10 nM concentration range with a 0.03 fM LOD. With their excellent sensitivity and low detection limit, these F-BN QDs enable new biological analysis methods for clinical diagnosis (Figure 5O).

Chen et al. [73] prepared NBF-CDs via a one-step hydrothermal method using malonate and the 1-allyl-3-vinyl imidazolium tetrafluoroborate ionic liquid as precursors (Figure 5P). NBF-CDs fluorescent probes based on the inner filter effect were developed for sulfathiazole detection. In these probes, the fluorescence intensity is linearly correlated with the sulfathiazole concentration within two ranges (0.008–10 and 10–45 μg/mL), with a 5.5 ng/mL LOD. Thus, confirming the use of this fluorescent probe in environmental applications.

Fluoride atoms can affect the optical properties and magnetism of CDs. However, when fluoride is not co-doped with other elements, QY tends to be low. Therefore, it is worthwhile to research the synthesize of F-doped CDs with high QY. Although fluoride is not prevalent in biological systems, F-containing grafts can be widely used in the biomedical field [74]. As shown in Table 1, while most of the doped F manifests as fluoride in the reaction medium, they also require sufficient expertise to be used safely. Ionic liquids are a more favorable alternative and often contain weakly coordinating fluoroanions, such as BF_4_^−^ or PF_6_^−^. Therefore, the synthesis of F-doped CDs based on ionic liquids is worth discussing.

### 3.2. Chlorine Doping

#### 3.2.1. Single-Chlorine Doping

**Figure 6:**
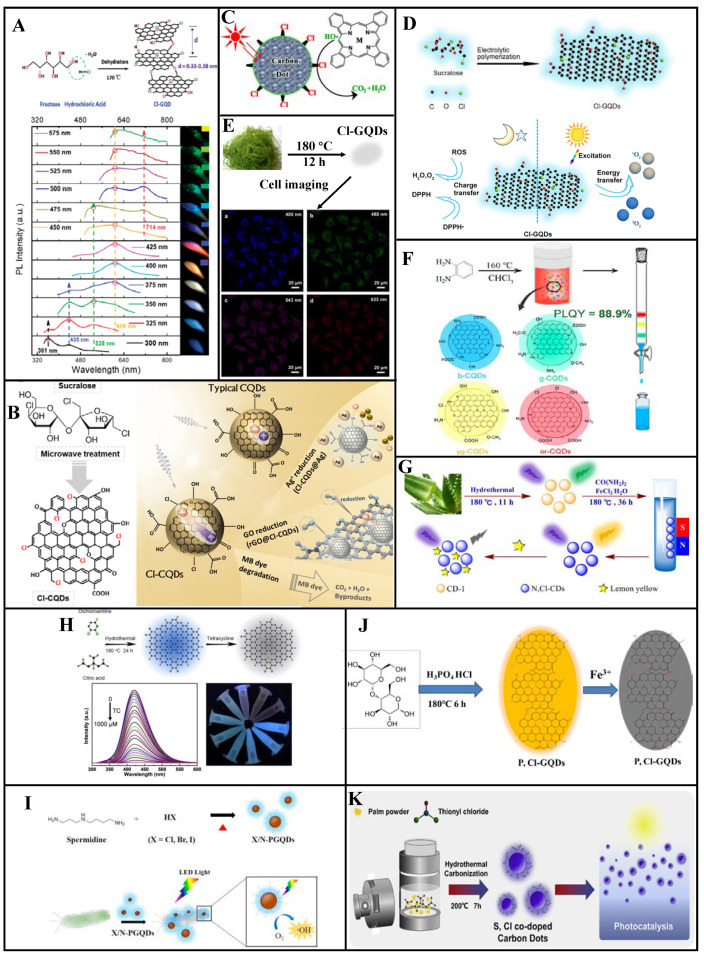
Synthesis and applications of Cl-doped CDs [79,83]. (**A**) Multicolor fluorescent Cl-GQDs were synthesized using fructose and hydrochloric acid as raw materials. (**B**) Microwave synthesis of Cl-CQDs by sucralose can be used for photocatalytic reduction and degradation of dyes. (**C**) Cl-CDs synthesized by the chlorine radical substitution reaction can rapidly photodegrade phenol cyanine. (**D**) The synthesis of Cl-GQDs by electrochemical oxidation showed good antioxidant activity. (**E**) Using seagrass as raw material, white Cl-GQDs were synthesized using the hydrothermal method for application in multicolor cell imaging. (**F**) Cl-CQDs were obtained by the solvothermal reaction of *o*-phenylenediamine under trichloromethane, and fluorescent Cl-CQDs in four colors were obtained by column separation. (**G**) N and Cl-CDs were synthesized from aloe vera, and tartrazine in beverages can be detected based on FRET. (**H**) The hydrothermal synthesis of NCl-GQDs using citric acid and 3,4-dichloroaniline allows for the highly sensitive detection of tetracycline. (**I**) Schematic of the synthesis pathway of X/N-PGQDs with excellent sterilization activity under visible light. (**J**) PCl-CQDs were obtained by hydrothermal synthesis of maltose, phosphoric acid, and hydrochloric acid, which can be used to detect Fe ions. (**K**) The S and Cl co-doped CDs derived from palm powder exhibit photocatalytic activity. Reprinted/adapted with permission from Ref. [75]. Copyright 2013, copyright RSC. Reprinted/adapted with permission from Refs. [75,77,80,81,82,84]. Copyright 2015, 2020 and 2021, copyright Elsevier. Reprinted/adapted with permission from [47,76,78]. Copyright 2019, 2021 American Chemical Society.

Chlorine doping of CDs can provide additional energy levels, yielding polychromatic fluorescence and tunable luminescence properties. Li et al. [75] prepared Cl-GQDs (QY: 6.8%, particle size: ~5.4 nm, calculated Cl-atom doping ratio: 2%) through hydrothermal treatment using fructose and hydrochloric acid (Figure 6A). Because Cl doping introduces additional energy levels between C π and C π* to create new electron transition pathways, these Cl-GQDs exhibit blue-to-red multicolor emission under excitation wavelengths of 300–600 nm. Multicolor Cl-GQDs are expected to be used in biological imaging and optoelectronic devices.

To overcome the limited light absorption outside the UV region and extremely fast photocarrier recombination of CQDs, Murali et al. [76] prepared Cl-CQDs from sucralose via microwave assisted synthesis. As shown in Figure 6B, Cl doping increased the absorption range and promoted the separation of the photoexcited carriers. Furthermore, the photodegradation efficiency was increased, which can cause rapid photodegradation of methylene blue. These Cl-CQDs show photochemical reduction activity toward Ag^+^ ions and graphene oxide via photoelectron transfer under visible-light irradiation.

Using CDs with surface oxygen-containing groups, Hu et al. [77] prepared Cl-CDs (particle size distribution: 2–5 nm, Cl-atom doping ratio: 2–3%, blue light emission) through the substitution reaction of Cl radicals into thionyl chloride molecules (Figure 6C). Compared to CDs with amine surface groups, these Cl-CDs exhibit considerably higher photocatalytic activity under visible-light irradiation and can even rapidly degrade phthalocyanines with high physical and chemical stability. Thus, these Cl-CDs have excellent metal-free photocatalytic performance and can be used as photocatalysts for organic pollutant degradation.

Cl-GQDs electrochemically synthesized by the Wang group exhibit both anti- and pro-oxidant activities [78]. The free radical scavenging and generation efficiency of these Cl-GQDs are seven and three times higher than those of un-doped GQDs, respectively. Moreover, under simulated sunlight irradiation, the Cl-GQDs demonstrate considerable antibacterial ability because of their enhanced singlet-oxygen production capacity (Figure 6D).

Li et al. [79] prepared Cl-GQDs (average particle size: 3.95 nm, QY: 28%) with white fluorescence by subjecting seaweed dispersed in a mixture of ethanol and chloroform to high-temperature, high-pressure treatment (Figure 6E). By adding an aqueous Cl-GQDs solution to HeLa cells, cell imaging revealed blue, green, purple, and red fluorescence under 405, 488, 543, and 633 nm excitation, respectively.

#### 3.2.2. Chlorine and Nitrogen Co-Doping

Zhang et al. [47] synthesized full-color-distribution NCl-CQDs in a one-step solvothermal process based on a co-doping strategy, using 1, 2-diaminobenzene and chloroform as raw materials (Figure 6F). They found that increased doped-atom particle size and content in the CQDs causes red-shifting of the PL spectrum, an increase in the π electron system, and decreased band gaps for the π→π* and n→π* transitions. Four different photoluminescent CQDs were determined via column chromatography, with 435, 495, 525, and 595 nm emission peaks corresponding to blue (QY: 40.7%), green QY: 88.9%), yellow-green (QY: 33.7%), and orange-red (QY: 34.8%) emissions, respectively. The researchers fabricated a flexible full-color emissive film by mixing the prepared NCl-CQDs into a polymer matrix. Four PVP/NCl-CQDs composite materials were each coated on separate 365/395 nm UV chips to yield multi-LED lamps with a maximum power efficiency of 17.38 lm/W. The multi-LED luminescence spectrum differed from those of the CQDs because of the polymerization of the CQDs surface functional groups in the thin films. An efficient white LED with a correlated color temperature of 4534 K, and a color rendering index of 90.8 was produced by adjusting the volume ratios of the four PVP/NCl-CQDs solutions.

Yang et al. [80] synthesized spherical NCl-CDs (average particle size: ~5 nm) via a hydrothermal method with urea, FeCl_3_·6H_2_O, and aloe as raw materials. A high QY of 60.52% was achieved (Figure 6G). Based on the Förster resonance energy transfer mechanism, the NCl-CDs are applicable to tartrazine detection, having a linear correlation within 0.1–30 μM for a 48 µM LOD.

Gu et al. [81] prepared NCl-GQDs via hydrothermal treatment of citric acid and 3, 4-dichloroaniline. These NCl-GQDs have strong blue fluorescence under 365 nm UV irradiation with a QY of 23.2% and several unique advantages, such as emission behavior independent of the excitation wavelength, high dispersity, and excellent stability. Moreover, a small amount of tetracycline quenches their fluorescence. As shown in Figure 6H, within 0.2–30 μM, good linearity occurs between the tetracycline concentration and the fluorescence quenching degree, with a 68 nM LOD. Moreover, their efficacy as tetracycline sensors has been confirmed by testing real water samples.

As shown in Figure 6I, Huang et al. synthesized Cl/N co-doped polymeric graphene quantum dots (Cl/N-PGQDs) from spermidine and hydrohalic acid by a simple one-step heating method (210, 240, and 270 °C) [82]. LED light irradiation of X/N-PGQDs promotes the generation of reactive oxygen species that, combined with their strong interaction with the bacterial membrane, cause cytoplasmic leakage and cell death. Among the three kinds of synthetic (X = Cl, Br, and I) X/N-PGQDs, Cl/N-PGQDs showed superior antibacterial activity.

#### 3.2.3. Chlorine and Phosphorus Co-Doping

Wang et al. [83] prepared PCl-CDs via a hydrothermal method using maltose as a precursor with phosphoric acid and hydrochloric acid (Figure 6J). Different electron transition paths were produced through bandgap formation due to the P and Cl co-doping, improving the QY (15%) of the CDs and increasing the fluorescence emission wavelength. These PCl-CDs have good dispersibility and photostability in aqueous solution and good Fe(III) determination in serum and water samples. Their fluorescence is gradually quenched by increasing Fe(III) concentration within 0.1–8.0 μM, with a 60 µM LOD. The fluorescence can be recovered by adding ethylene diamine tetraacetic acid. Hence, these PCl-CDs can be used as sensors in medicine, food, and environmental applications.

#### 3.2.4. Chlorine and Sulfur Co-Doping

Zhu et al. [84] synthesized SCl-CDs (average particle size: 3.54 ± 0.82 nm, QY: 0.9%) through a hydrothermal approach using thionyl chloride as a dopant and palm powder as the carbon source (Figure 6K). These SCl-CDs exhibit excitation-dependent PL behavior with a 425 nm maximum emission wavelength. The self-absorption behavior manifests as an overlap of the excitation, and UV absorption spectra and significantly reduces the QY. Under visible light, the SCl-CDs exhibit apparent charge separation. The reactive radicals produced by the reaction between the oxidation/reducing agent and the electrons or holes trapped at various SCl-CDs surface defect sites have photocatalytic degradation activity toward organic dyes, with photocatalytic degradation efficiencies of 94.2% and 71.7% for methylene blue and rhodamine B, respectively.

Chlorine doping is an effective method to improve the PL, catalytic efficiency, antioxidant and pro-oxidation performance, and antibacterial activity of CDs. As an electron-rich species, Cl exhibits strong electronegativity and when doped into CDs and provide additional electronic energy levels, thus producing polychromatic fluorescence and tunable PL. As shown in Table 2, Cl-CDs are well-suited to the fields of photocatalysis and sensing. Hence, it can be argued that its anti-oxidation, pro-oxidation, and bactericidal properties merits further study. The synthesis of elemental doped CDs by radical substitution is an interesting direction.

### 3.3. Bromine Doping

#### 3.3.1. Single-Bromine Doping

**Figure 7:**
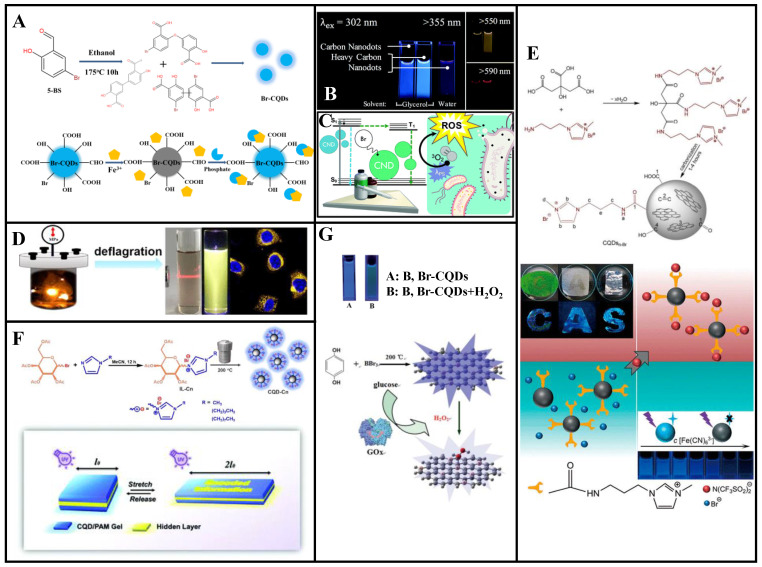
Synthesis and applications of Br-doped CDs. (**A**) Br-CQDs were synthesized with 5-BS as the precursor, and its fluorescence was quenched with Fe ions. Phosphate can be added to restore fluorescence. (**B**) By doping bromine atoms, CDs exhibit room-temperature phosphorescence via the heavy-atom effect. (**C**) Br-doped CDs exhibit Type I and Type II photosensitization under UVA irradiation via the heavy-atom effect and possess excellent antibacterial properties. (**D**) NBr-CQDs were synthesized using the deflagration method for cell imaging. (**E**) Synthesis and application of ionic liquid-modified CQDs. (**F**) Schematic of the imidazole ionic liquid NBr-doped CQDs synthesis and its use in information encryption. (**G**) Schematic of the BBr-CQD synthesis, which can be quenched by hydrogen peroxide. The quenching reaction can be used to detect Gox activity. Reprinted/adapted with permission from Refs. [86,87,90,91]. Copyright 2014, 2018, 2020, and 2021 copyright RSC. Reprinted/adapted with permission from [88,89]. Copyright 2015, 2021 American Chemical Society.

Bromine-doped CDs can be applied to environmental monitoring and biological imaging. Zou et al. [85] synthesized Br-CDs with blue fluorescence (QY: 25%) via hydrothermal treatment using 5-bromosalicylaldehyde (5-BS). These Br-CDs have a narrower particle size distribution than undoped CDs and good hydrophilicity, pH stability, high photostability, and low cytotoxicity. The UV absorption spectrum of an absorbent formed using the Br-CDs and Fe^3+^ exhibit partially overlapped excitation and emission profiles, indicating that the Br-CDs fluorescence was quenched by Fe^3+^ through the inner filter effect. The quenched fluorescence could be restored to 88% of the original value by adding phosphate. The fluorescence intensity of a composite Br-CDs/Fe^3+^ nanoprobe was highly linearly correlated with the phosphate concentration within 0.4–22 μM, with a 0.25 μM LOD. The precision for five repeated tests was 3.6%, confirming the reliability of the Br-CDs/Fe^3+^ phosphate testing (Figure 7A). Thus, these Br-CDs have good selectivity as fluorescent probes for ultrasensitive fluorescence-quenching-based Fe^3+^ detection and intracellular phosphate detection through confocal imaging. Therefore, these Br-CDs have considerable potential for environmental monitoring, intracellular imaging, and other biomedical applications.

The heavy-atom effect refers to the phenomenon wherein atoms with large atomic numbers enable (or enhance the existing) spin-orbital coupling of molecules, which increases the probability of S_0_ → T_1_ absorption transition and S_1_ → T_1_ system hopping. This is beneficial for phosphorescence generation [86]. To synthesize CDs with multicolor fluorescence and room-temperature phosphorescence, Knoblauch et al. [87] doped Br into CDs, which stimulated their transition from the singlet to the triplet state, demonstrated phosphorescence through internal heavy-atom effect, and confirmed that acidity was an important condition for the formation of phosphorescent CDs (Figure 7B). In their follow-up work, the authors harnessed the heavy-atom effect and exposed the prepared Br-CDs to UV-A radiation, exhibiting significant antibacterial effect (Figure 7C) [86].

#### 3.3.2. Bromine and Nitrogen Co-Doping

Gong et al. [88] first designed a deflagration method for the rapid synthesis of NBr-GQDs (average diameter: 3.5 nm, thickness: <2 nm) that emit yellow fluorescence owing to their PL (Figure 7D). Because of the N and Br co-doping, the band gap between the excited singlet states and their crossover with triplet states are reduced; hence, the GQDs exhibit a high QY (52%). These NBr-GQDs have good pH stability, long fluorescence lifetime, and low cytotoxicity and exhibit high-quality fluorescence labeling, showing great potential for biological imaging.

Wang et al. [89] prepared NBr-CQDs (particle size distribution: 0.6–1.6 nm, QY: up to 25.1%) with high charge densities through one-pot pyrolysis using citric acid and 1-aminopropyl-3-methyl-imidazolium bromide as precursors (Figure 7E). These NBr-CQDs are stable in various environments and can be used as fluorescent ink and fluorescence sensors for the detection of Fe (CN)_6_^3−^ and Fe (CN)_6_^4−^. Interestingly, the CQDs amphiphilicity can be tuned and amplified via anion exchange and phase transfer.

Wu et al. [90] prepared NBr-CQDs based on an imidazole ionic liquid. Different carbon chain lengths and anion types were used to regulate the CQDs hydrophilic and lipophilic properties (Figure 7F). With increased carbon-chain lengths of the cation, the CQDs QY gradually increased from 5.7% to 8.8% and 10.5% (corresponding to carbon chain lengths of 1, 4, and 8), respectively. Hydrophobic or hydrophilic NBr-CQDs gels were prepared by tuning different physical properties. A sandwich-type mechanical color-changing device was designed, which contained NBr-CQDs gels as outer layers. Encoded information was printed on the bottom layers in fluorescent inks. This secret information was invisible under visible light but gradually became visible when the gel was stretched under a UV lamp. Thus, this NBr-CQDs gel has a dual response mechanism to light and mechanical force, rendering it more secure than traditional single-encryption ink for information encryption.

#### 3.3.3. Bromine and Boron Co-Doping

Shan et al. [91] prepared BBr-CQDs with 5.9% B and 3.6% Br contents by weight via a solvothermal method using hydroquinone and BBr_3_ (QY: 14.8%). These BBr-CQDs can be used as fluorescence sensors for H_2_O_2_ and glucose detection (Figure 7G). The fluorescence quenching efficiency is linearly correlated with the H_2_O_2_ concentration from 0.1 to 1.0 mM, and the fluorescence can be recovered through the addition of MnO_2_. Neither glucose oxidase (GOx) nor glucose can quench the BBr-CQDs fluorescence, which is largely quenched by their combination. At pH 7.4, a BBr-CQDs/GOx fluorescence sensor can be employed for glucose detection. The fluorescence quenching efficiency and glucose concentration are linearly correlated within 8.0–80.0 mM, for an 8.0 mM LOD.

Bromine-doping introduces a new surface state to promote this aspect, consisting of a reversible “on-off-on” fluorescence-sensing system. Moreover, the combined use of a carbon source and Br-containing ionic liquid yielded positively-charged Br-CDs which, compared with existing CDs, were found to possess novel characteristics and superior properties, such as stability under various environments and amphiphilicity. Furthermore, co-doping of Br and N improves the luminescence properties of CDs. Due to the heavy-atom effect, room-temperature phosphorescence induced by the incorporation of Br into CDs is a promising research direction. In addition, the outstanding QY of Br-CDs prepared by deflagration method warrants further investigation (Table 3).

### 3.4. Iodine Doping

#### 3.4.1. Single-Iodine Doping

**Figure 8:**
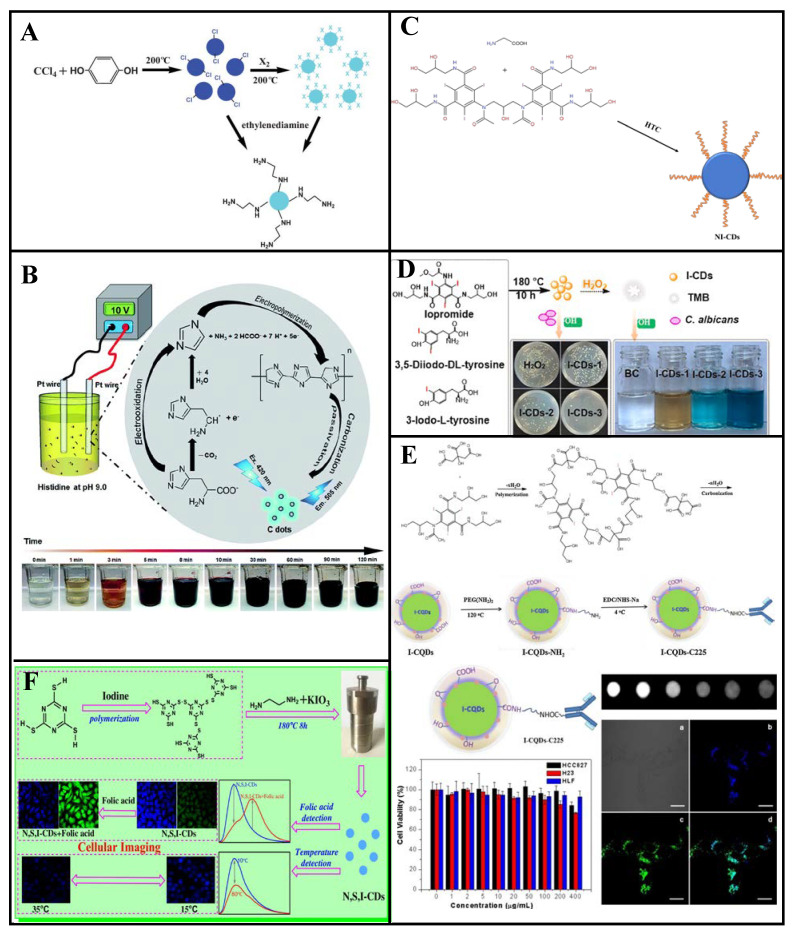
Synthesis and applications of I-doped CDs. (**A**) Solvothermal synthesis of Cl-CQDs as the intermediates and I_2_ as the modifier to convert the Cl-CQDs into I-CQDs; both CQDs can function as intermediates. (**B**) Schematic of I-CQDs preparation by electrolysis. (**C**) Hydrothermal synthesis of NI-CDs by iodixanol and glycine can be used as a cell imaging and CT contrast agent. (**D**) NI-CDs synthesized using ethylenediamine and 3-iodo-L-tyrosine, 3,5-diiodo-DL-tyrosine, or iopromide exhibit photocatalytic antibacterial activity in the presence of hydrogen peroxide. (**E**) NI-CDs were synthesized via the hydrothermal method with citric acid and iodohexanol as precursors for application as fluorescent CT bimodal imaging probes. (**F**) NSI-co-doped CDs were prepared for application in folic acid and temperature sensing, and cellular imaging. Reprinted/adapted with permission from Refs. [57,92]. Copyright 2013, 2019 copyright RSC. Reprinted/adapted with permission from Refs. [95,96,97]. Copyright 2018, 2020 and 2021, copyright Elsevier.

Zhou et al. [57] synthesized Cl-CQDs using carbon tetrachloride and hydroquinone as raw materials at 200 °C and obtained I-CQDs through the substitution reaction between the Cl-CQDs and I_2_ (Figure 8A). The I-CQDs particle size was independent of the I_2_ treatment, and their maximum emission wavelength was 435 nm (QY: 1.7%) and prominent fluorescence under a UV lamp. These I-CQDs can be used as intermediates for N-CQDs synthesis with 1, 2-ethylenediamine-modified surface functional groups.

The Lin group reported a synthesis of I-CDs by adding NaI to salt acidified histidine under a 10 V applied voltage (Figure 8B) [92]. These I-CDs are susceptible to cross quenching, and their fluorescence intensity decreases with increasing Cu^2+^, Hg^2+^, and Ag^+^ concentration. At 0.8 mm iodide concentration, the I-CDs have better Cu^2+^ selectivity. Good linearity between the I-CDs and Cu^2+^ occurs within 0.3–3 μM, with a 0.22 μM LOD. These I-CDs are effective Cu^2+^ fluorescence sensors, as demonstrated through testing of various real water samples.

#### 3.4.2. Iodine and Nitrogen Co-Doping

Iodine-doped CDs have considerable potential for computed tomography (CT) imaging, biological imaging, and bacteriostasis because of the high atomic number and unique properties of iodine. Zhang et al. [93] reported bottom-up hydrothermal synthesis of NI-CDs (average particle size: 2.7 nm, maximum emission wavelength: 475 nm) for use as a CT contrast agent and fluorescent probe, with iodixanol and glycine as dopants (Figure 8C). These NI-CDs possess new physicochemical properties and have attracted considerable attention because of their excellent fluorescence properties, dispersibility, stability, and good biocompatibility. In addition, iodine endows them with strong X-ray attenuation ability. Moreover, NI-CDs can be effectively excreted through renal clearance after systematic administration, signposting potential medical applications. Thus, NI-CDs have considerable application potential as bi-modal probes for biomedical research and disease diagnosis.

Wang et al. [94] synthesized NI-CDs (QY: 37%) through a one-step hydrothermal method, which exhibit peroxidase-like activity and can catalyze H_2_O_2_ to generate a hydroxyl radical (•OH) under visible light; hence, the reactive oxygen species level is improved. These NI-CDs exhibit strong photocatalytic antibacterial activity against *Escherichia coli* and *Staphylococcus aureus* in the presence of H_2_O_2_, and significant bacterial infection prevention and healing acceleration of artificial wounds in the presence of ultra-low H_2_O_2_ concentrations. Thus, NI-CDs have considerable application potential owing to their high antibacterial efficiency, low toxicity, and good biocompatibility. These advantageous characteristics promote further development of nanomaterial enzymes for wound healing and treatment of other diseases.

Li et al. [95] synthesized NI-CDs via hydrothermal treatment using ethylenediamine and 3-iodo-L-tyrosine, 3, 5-diiodo-DL-tyrosine, or iopromide as distinct iodine sources (Figure 8D). The NI-CDs synthesized using iopromide achieve antifungal disinfection toward *Candida albicans* in the presence of exogenous H_2_O_2_, with performance similar to that of peroxidase. The peroxidase-like and antifungal activities of the NI-CDs gradually increase with increasing iodine content. These NI-CDs have more than 90% antifungal activity against *C.* *albicans* under visible light for 120 min in the presence of H_2_O_2_, which is attributed to the synergistic effect of peroxidase and photocatalytic activities.

Using citric acid and iohexol as precursors, Su et al. [96] synthesized NI-CQDs (QY: 18%) via a hydrothermal method that NI-CQDs are applicable as probes for fluorescence CT bimodal imaging. The CQDs were conjugated with a target molecule (Cetuximab) to facilitate CQDs-C225 labeling of HCC827 (lung cancer cell line, epidermal growth factor receptor (EGFR) over expression), H23 (lung cancer cell line, EGFR under expression), and HLF (normal lung cell line, EGFR under expression) cells. The HCC827 cells were labeled with strong fluorescence, suggesting that CQDs-C225 can specifically target EGFR-overexpressed cancer cells through EGFR-mediated endocytosis, with combined advantages of high sensitivity and good spatial resolution. These NI-CQDs are excellent targeting materials for cancer cell labeling and the transport of drugs into targeted cancer cells. Thus, they have considerable potential for future application to cancer treatment and biological imaging (Figure 8E).

#### 3.4.3. Nitrogen, Sulfur, and Iodine Co-Doping

CDs doped with iodine and an appropriate element can exhibit interesting temperature-dependent emission phenomena. For example, Mu et al. [97] prepared NSI-CDs (QY: 32.4%) through a hydrothermal reaction using C_3_N_3_S_3_ and ethylenediamine as precursors and potassium iodate as an additive (Figure 8F). The N, S, and I co-doping redshifted the maximum emission wavelength in the presence of folic acid and was accompanied by weak fluorescence quenching. These NSI-CDs exhibit temperature-dependent emission at 10–80 °C and temperature sensing behavior for imaging of human colon cancer cells (HT-29), proving to be good cell imaging agents. Therefore, these synthetic NSI-CDs have broad development prospects for biomedical and environmental applications.

As an effective fungicide, iodine is the only element that can destroy microorganisms without contaminating the environment [98]. Most of the germicidal ability of I-CDs is attributed to peroxidase activity via the catalysis of H_2_O_2_ to produce reactive oxygen for germicide; however, the underlying mechanism remains unclear. At the same time, due to the high atomic number of iodine, I-CDs exhibit good X-ray attenuation ability, which can be applied to CT imaging and other imaging applications. However, methods for their synthesis are insufficient and their QY are not as high as those obtained from hydrothermal synthesis (Table 4). The development of novel methods for the synthesis of I-CDs with improved QY merits further study.

## 4. Conclusions and Prospects

This review summarized halogen-doped preparation methods of CDs and their applications in various fields. Halogen doping of CDs is an effective approach in enhancing their optical and physicochemical properties. In particular, the application of halogen-doped CDs to biological imaging and sensing has been studied intensively. However, their application to photocatalytic degradation, multifunctional electrodes, gene transfection, gene detection, CT imaging, and ECL biosensing has not been widely reported to date. In the context of biological imaging, studies have thus far shown that most halogen-doped CDs can only be used to label the cell membrane and cytoplasm, but not the nucleus. Moreover, questions remain regarding, e.g., the structures of halogen-doped CDs, their formation mechanisms, and the effects of halogen doping on the optical and other properties of the CDs.

As part of the ongoing exploration of CDs, their practical applications have expanded. However, their preparation and widespread adoption still face the following limitations. (1) Their synthesis using various raw materials has endowed CDs with diverse properties. However, the associated microstructures, mechanism of luminescence, and formation process remain unclear. (2) CDs with long wavelength emission have immense potential for application in the fields of biological imaging and file encryption. However, due to the limitations of their synthesis and a lack of sufficient clarity regarding their luminescence mechanism, efficient water-soluble CDs with long wavelength emissions currently remain scarce. Therefore, the synthesis and characterization of efficient water-soluble CDs with long and controllable wavelength emissions needs to be studied in future works. (3) CDs prepared via existing methods cannot achieve precise heteroatom doping, such as doping at the edge or center of the CDs. Therefore, it is of great significance to develop standardized methods for CDs synthesis, purification, and accurate structural characterization. (4) The precision of the heteroatom doping ratio needs further improvement. (5) CDs have been widely studied in the fields of fluorescent probes and biological imaging. These CDs are highly selective and sensitive to stimuli, exhibit fast response times, and are low-cost. Despite these advantages, their application is still in the laboratory stage and further improvements in stability, selectivity, and anti-jamming capabilities are required. Improving these aspects and up-scaling to practical applications should be prioritized. In short, CDs possess several advantages that other quantum dots do not have, and thus have huge application potential in various fields. At present, research into CDs is focused on their simple preparation and convenient purification. It is believed that, with further research, the limitations of CDs will be solved one by one.

## Figures and Tables

**Table 1:** Synthesis methods, raw materials, and applications of F-CDs.

Method	Raw Material	Elements	QY (%)	Application	Ref.
Solvothermal	3, 4-difluorophenylhydrazine	F, N	16.9	Detection of cytochrome C	[68]
	3,3,4,4,5,5,6,6,7,7,8,8,8-tridecafluoro-1-octanol and poly ethylene imine		––	Drug delivery	[66]
	4,5-difluorobenzene-1,2-diamine and tartaric acid		31 and 14	Cell imaging Detection of Ag^+^	[62]
	Citric acid, 3-fluoroaniline	N, F, S	3.17	Temperature sensing	[71]
Hydrothermal	Sodium fluoride, urea, and citric acid	F, N	15.1	Antioxidant Cell imaging Better electrical performance	[64]
	Malonate and 1-allyl-3-vinyl imidazolium tetrafluoroborate ionic liquid	N, B, F	––	Detection of sulfathiazole	[73]
Microwave	Ionic liquid, multi-walled carbon nanotubes	N, F, S	70	––	[70]
Microwave-assisted hydrothermal	Glucose, HF	F	––	Prevent hIAPP aggregation	[60]
	Glucose, levofloxacin	F, N	––	MRI contrast agents	[67]
	Fluorinated graphene oxide (FGO) nanosheets, HNO3		7.5	Cell imaging	[63]
	H_3_BO_3_, C_3_H_6_N_6_ and NH_4_F	N, B, F	––	Detection of K-ras	[72]
Surface passivation	Tetrafluoroterephthalic acid and N-CDs	F, N	58.9	Detection of 4-NP	[61]
Gas-phase fluorination	F_2_ and N-CDs	F, N	8.3	Information storage security	[69]
Ring-opening polymerization and dehydration carbonization	Polyethyleneimine and fluorinated diglycidyl ether	F, N	––	Aggregation of DNA	[65]
Pyrolysis	Fluorinated graphene	F	6.3	Strong paramagnetism	[59]

**Table 2:** Synthesis methods, raw materials, and applications of Cl-CDs.

Method	Raw Material	Elements	QY (%)	Application	Ref.
Microwave-assisted hydrothermal	Sucralose	Cl	––	Photochemical reduction of Ag^+^, Dye degradation, photoelectron transfer of GO	[76]
Substitution reaction of Cl radicals	CDs with surface oxygen-containing groups	Cl	––	photocatalysts for organic pollutant degradation	[77]
Electrochemically	Sucralose	Cl	––	anti- and pro-oxidant, antibacterial	[78]
Pyrolysis	Spermidine and HX	N, Cl	3.85–6.13%	Photocatalytic bacteriostasis	[82]
Solvothermal	seaweed	Cl	28	White fluorescent, Cell imaging	[79]
	1, 2-diaminobenzene, chloroform	N, Cl	Blue 40.7, Green 88.9, Yellow 33.7, Orange 34.8	Multicolor LEDs	[47]
Hydrothermal	Fructose, hydrochloric acid	Cl	6.8	––	[75]
	Urea, FeCl_3_·6H_2_O, and aloe	N, Cl	60.52	Detection of tartrazine	[80]
	Citric acid and 3, 4-dichloroaniline		23.2	Detection of tetracycline	[81]
	Maltose, phosphoric acid, and hydrochloric acid	P, Cl	15	Detection of Fe^3+^	[83]
	Thionyl chloride and palm powder	S, Cl	0.9	Dye degradation	[84]

**Table 3:** Synthesis methods, raw materials, and applications of Br-CDs.

Method	Raw Material	Elements	QY (%)	Application	Ref.
Hydrothermal	5-BS	Br	25	Detection of Fe^3+^, Detection of intracellular phosphate	[85]
	Citric acid and 1-aminopropyl-3-methyl-imidazolium bromide	N, Br	25.1	Detection of Fe (CN)_6_^3−^ or Fe (CN)_6_^4−^, Fluorescent ink	[89]
	Imidazole ionic liquid		5.7 (C-1) 8.8 (C-4) 10.5 (C-8)	Encryption ink	[90]
Deflagration	C_6_Br_6_ and NaN_3_	N, Br	52	Cell imaging	[88]
Solvothermal	Hydroquinone and BBr_3_	B, Br	14.8	Detection of H_2_O_2_ and glucose	[91]

**Table 4:** Synthesis methods, raw materials, and applications of I-CDs.

Method	Raw Material	Elements	QY (%)	Application	Ref.
Surface passivation	I_2_ and Cl-CQDs	I	1.7	Intermediates for N-CQDs synthesis	[57]
Electrochemically	Salt acidified histidine		––	Detection of Cu^2+^	[92]
Hydrothermal	Iodixanol and glycine	N, I	––	Cell imaging, CT imaging	[93]
	Iohexol		37	Antibacterial, Wound healing	[94]
	Ethylenediamine and 3-iodo-L-tyrosine, 3, 5-diiodo-DL-tyrosine, or iopromide		––	Photocatalytic antibacterial	[95]
	Citric acid and iohexol		18	Fluorescence CT bimodal imaging	[96]
	C_3_N_3_S_3_ and ethylenediamine and potassium iodate	N, S, I	32.4	Cell imaging, Temperature sensing	[97]

## Data Availability

Not applicable.
